# Data on overlapping brain disorders and emerging drug targets in human Dopamine Receptors Interaction Network

**DOI:** 10.1016/j.dib.2017.04.001

**Published:** 2017-04-08

**Authors:** Avijit Podder, N. Latha

**Affiliations:** Bioinformatics Infrastructure Facility, Sri Venkateswara College (University of Delhi), Benito Juarez Road, Dhaula Kuan, 110021 New Delhi, India

**Keywords:** Dopamine receptors, Interactions network, Brain disorders, Drug targets

## Abstract

Intercommunication of Dopamine Receptors (DRs) with their associate protein partners is crucial to maintain regular brain function in human. Majority of the brain disorders arise due to malfunctioning of such communication process. Hence, contributions of genetic factors, as well as phenotypic indications for various neurological and psychiatric disorders are often attributed as sharing in nature. In our earlier research article entitled “Human Dopamine Receptors Interaction Network (DRIN): a systems biology perspective on topology, stability and functionality of the network” (Podder et al., 2014) [1], we had depicted a holistic interaction map of human Dopamine Receptors. Given emphasis on the topological parameters, we had characterized the functionality along with the vulnerable properties of the network. In support of this, we hereby provide an additional data highlighting the genetic overlapping of various brain disorders in the network. The data indicates the sharing nature of disease genes for various neurological and psychiatric disorders in dopamine receptors connecting protein-protein interactions network. The data also indicates toward an alternative approach to prioritize proteins for overlapping brain disorders as valuable drug targets in the network.

**Specifications Table**

**Table:** 

Subject area	*Computational Systems Biology*
More specific subject area	*Human Dopamine Receptors Network*
Type of data	*Text files, tables and figures*
How data was acquired	*Systemic database curation and statistical analysis*
Data format	*Analyzed*
Experimental factors	*Common genetic factors responsible for various brain disorders in the network*
Experimental features	*Comprehensive mapping of disease genes and identification of overlapping disease modules in the network*
Data source location	*n/a*
Data accessibility	*Data are within this article*

**Value of the data**

• The data hints an overlapping disease spectrum of neurological disorders in DRIN.
• The data pinpoint towards common contributing genetic factors of neurological disorders in DRIN.
• The data might encourage researchers to identify additional drug targets in DRIN for future drug discovery endeavors.

## 1 Data

**Fig. 1:**
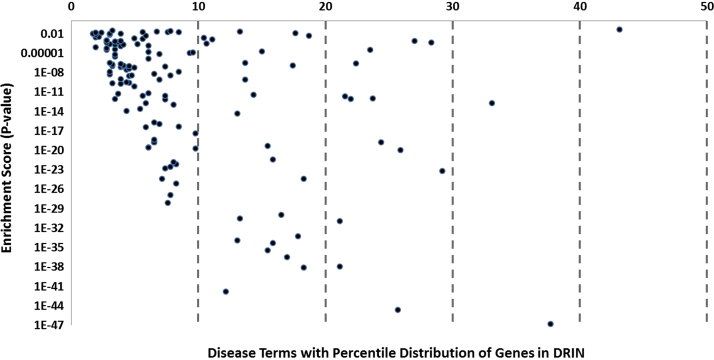
Gene-disease enrichment analysis in DRIN. In the plot the *X*-axis represents the percentage of genes associated with each disease terms in DRIN and *Y*-axis represents the enrichment statistics (*P*-value cut off <0.05) of disease terms in DRIN. A total of 143 different disease terms were significantly identified (blue dots) over 4312 disease association in DRIN after bonferroni correction of the *P*-value (two-sided hypergeometric test).

**Fig. 2:**
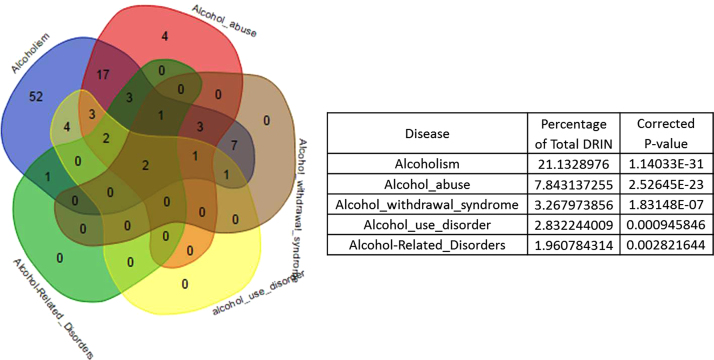
Related disease terms as a single disease module. Linked disease terms were symbolized under a single disease head to avoid overrepresentation of a disease in DRIN. As for example, the disease term “*Alcoholism*” can clearly represent five different alcohol related disease terms as a single disease module in the DRIN. Total 29 distinct disease modules were identified from 143 disease terms considering *P*-value cut off <0.05.

**Fig. 3:**
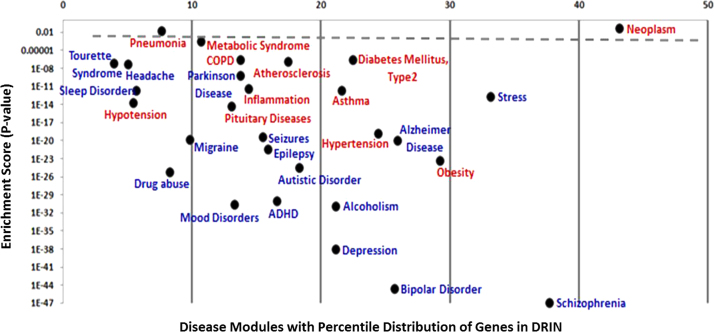
Disease Modules Enrichment in DRIN. A total of 29 disease modules related to brain disorders (blue) and not related to brain disorders (brown) are highlighted separately in the plot. The *X*-axis represents the percentage of genes associated with each disease module in DRIN and the *Y*-axis represents the enrichment statistics (*P*-value cut off <0.05) of disease modules in DRIN.

**Fig. 4:**
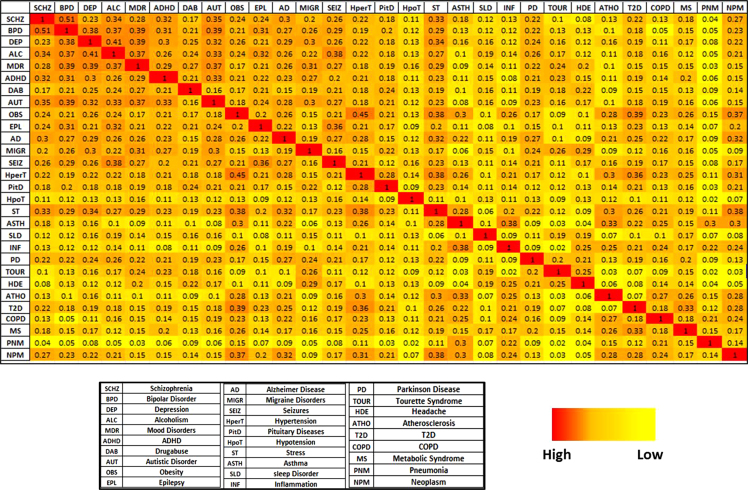
Overlapping disease modules in DRIN. The heat map showed an overlapping ratio of genes for 29 different disease modules in DRIN. The ratio equals to zero (yellow) if there are no intersecting genes and equals to one (red) if all gene intersect to disease modules.

**Fig. 5:**
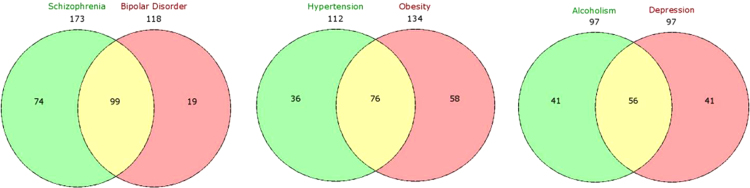
Pair wise comparison of overlapping disease modules in DRIN. Number of shared genes highlighted (yellow) for top three pairs of overlapping disease modules in DRIN.

**Fig. 6:**
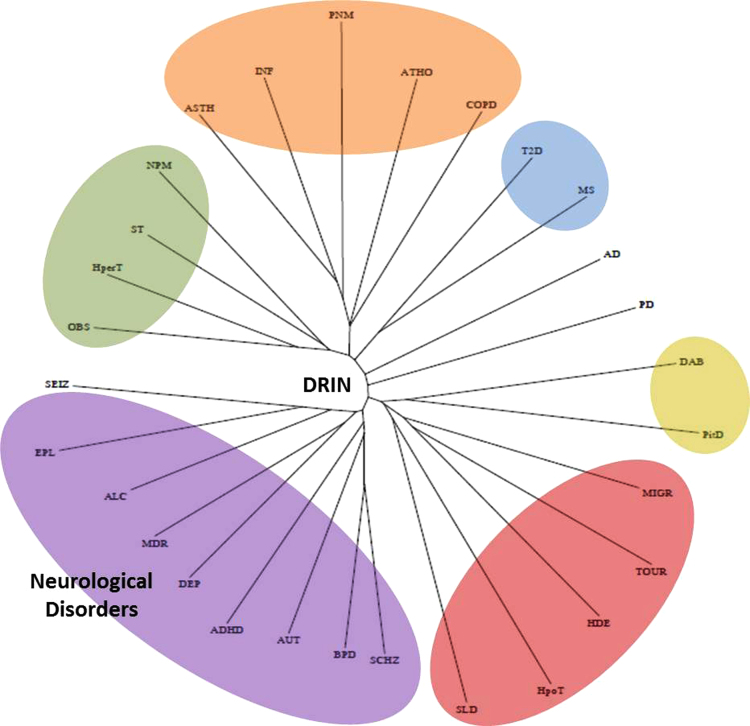
Disease modules clustering based on similarity and diversity of gene sets. A radial tree view highlighting different disease clusters for 29 disease modules. The largest branch in the tree (purple) holds all the disease modules related to neurological disorders in DRIN.

**Fig. 7:**
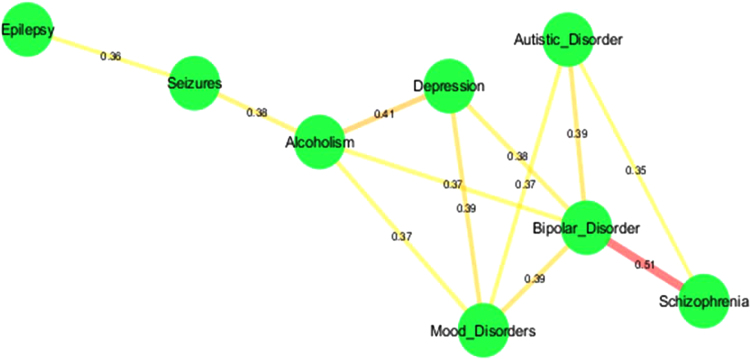
Connectivity map within disease modules in neurological disorder cluster. Disease modules are connected to each other based on the overlapping ratio cutoff <0.35 as derived from Jaccard or Tanimoto coefficient metrics.

**Fig. 8:**
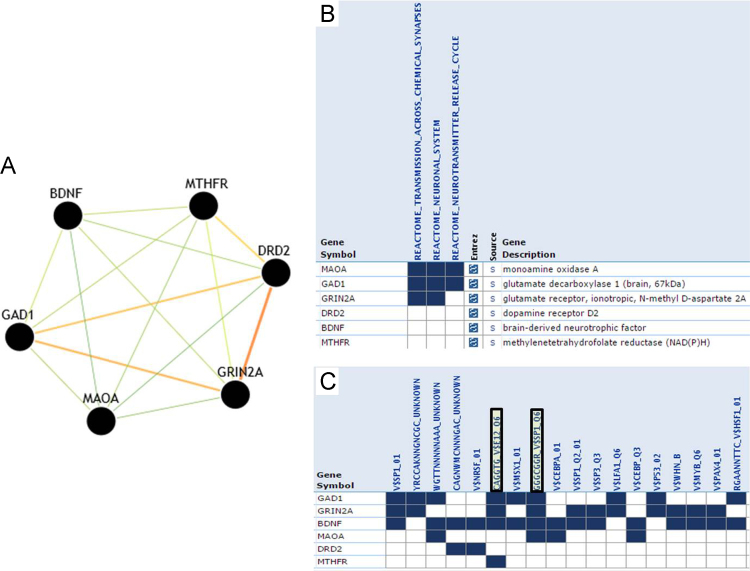
Common set of genes within disease modules in neurological disorder cluster. Panel [A] highlights six genes (*BDNF, DRD2, GAD1, GRIN2A, MAOA* and *MTHFR*) which are common contributing factors for all eight disease modules in neurological disorder cluster (Fig. 7). Panel [B] shows pathway enrichment and functional classification of genes in DRIN. Panel [C] depicts Transcription Factors (TFs) binding information highlighting regulatory classification of genes in the network.

**Fig. 9:**
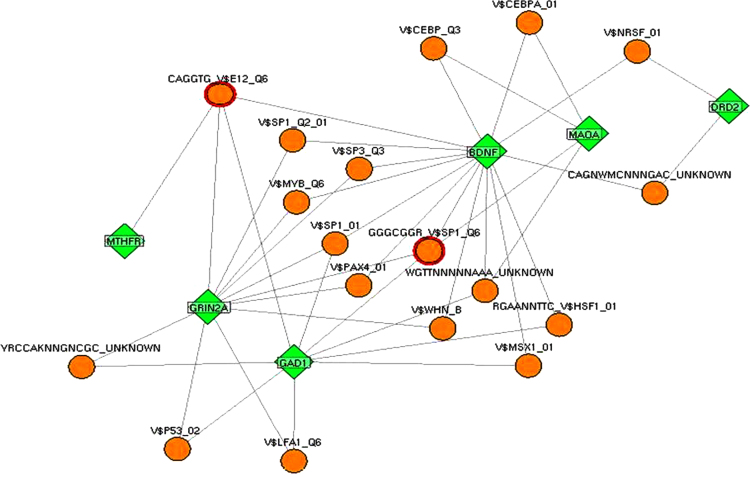
Transcription Factors (TFs) Regulatory network of disease genes in DRIN. The gene-TF interactions network exhibited TFs for which regulatory information from experimental data is available in the database. TFs highlighted are common for majority of the genes in the network.

**Fig. 10:**
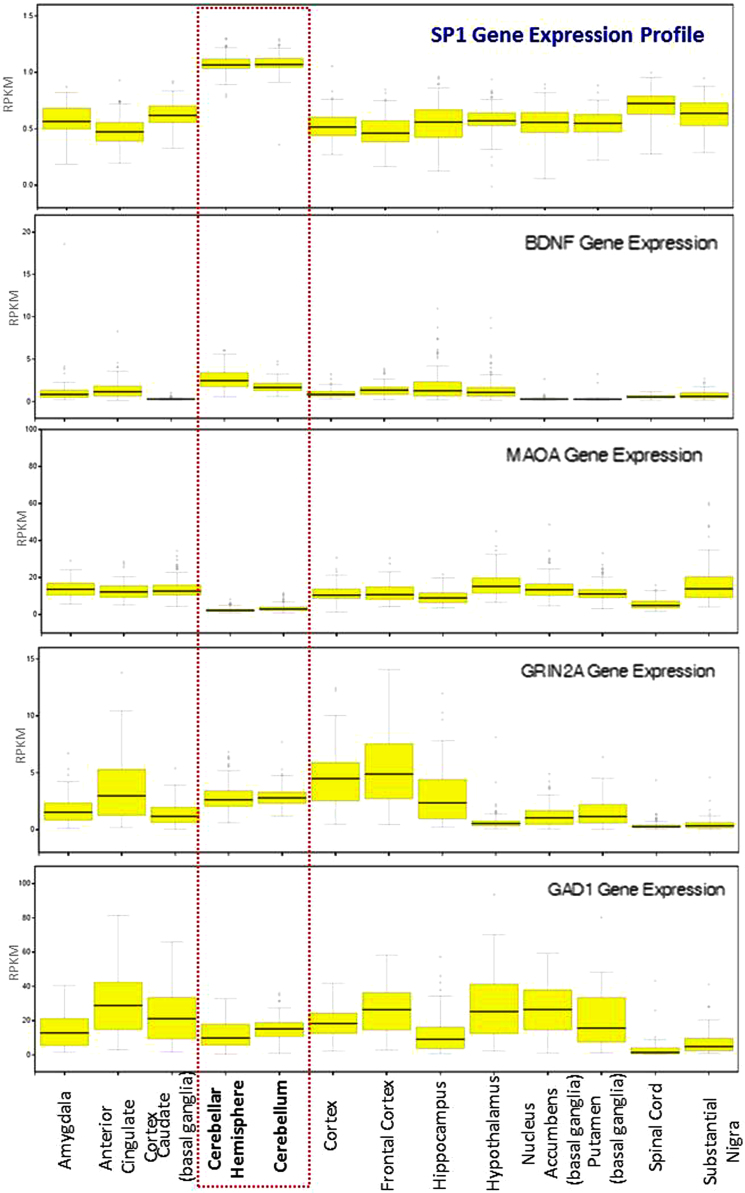
Tissue specific expression profiling of genes regulated by TF-Sp1 in human brain. Expression profiling of four genes and TF-SP1 in 13 different human brain tissues under normal physiological condition. Gene expression data were implemented through GTEx portal web server [8] where expression values are shown in RPKM (Reads Per Kilobase of transcript per Million mapped reads). Box plots are shown as median and 25th and 75th percentiles; points are displayed as outliers if they are above or below 1.5 times the interquartile range.

**Fig. 11:**
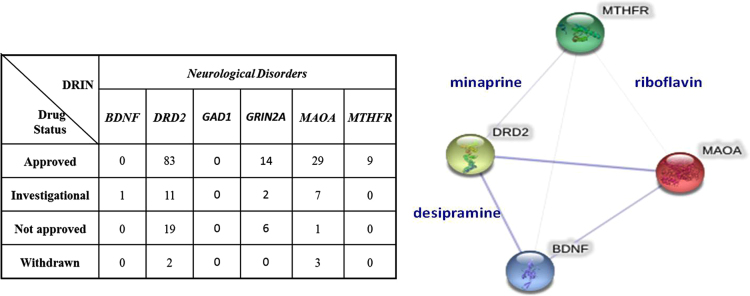
Drug availability of targeted proteins in DRIN. A classification of drug information from the combined data of DrugBank database and a study by Rask-Andersen [10] highlighted the number of approved, investigational, not approved and withdrawn drugs for all six different targets in DRIN. Drugs that are common for more than one target [minaprine (DB00805), desipramine (DB01151) and riboflavin (DB00140)] are also shown through an interactive diagram of the data.

The data highlighted 25848 associations between 431 proteins and 4312 diseases, disorders and clinical or abnormal human phenotypes in Dopamine Receptors Interactions Network (DRIN) [1]. The associations were refined through stringent statistical analysis to reduce the over representation of disease information in DRIN (Table S1). Further, related disease terms were combined together and significant associations of 29 disease modules in the DRIN were obtained (Fig. 1, Fig. 2, Fig. 3). The disease modules were classified based on the distributions of overlapping genetic factors (Fig. 4, Fig. 5, Fig. 6, Fig. 7). In addition, the common contribution factors for different brain disorders were characterized based on functional enrichment analysis to pinpoint additional drug targets in human DRIN (Fig. 8, Fig. 9, Fig. 10). Data were also tabulated to get more insights into drug availability for those targets (Fig. 11).

## 2 Experimental design, materials and methods

### 2.1 Disease enrichment data analysis

Genes were mapped to respective disease data using DisGeNet database [2]. The disease data indicates an association of diseases, disorders and clinical or abnormal human phenotypes with a given set of gene list. The data covers different biomedical aspects of each disease from several sources.

To identify significant enrichment of diseases in our data two-sided hypergeometric test [3] were carried out. The *P*-values were further corrected by the bonferroni correction (*P*≤0.05) to determine a list of significant disease association in our data.

$$P\left(X=x\right)=f\left(x\right)=\frac{\left(m/x\right)\left(N-m/n-x\right)}{\left(N/n\right)}$$

where *N*=Total number of genes in DisGeNet; *n*=Total number of genes in DRIN; *m*=Each disease associated genes in DisGeNet; *x*=Each disease associated genes in DRIN

### 2.2 Disease overlapping data analysis

Overlapping of genes between two diseases was measured using Jaccard or Tanimoto coefficient metrics [4] which compared the similarity and diversity of disease sets in our data. It uses the ratio of the intersecting set to the union set as the measure of similarity. Thus it equals to zero if there are no intersecting elements and equals to one if all elements intersect.

$$T=\frac{N_{c}}{N_{a}+N_{b}-N_{c}}$$

where *N*_a_=number of elements in set A; *N*_b_=number of elements in set B; *N*_c_=number of elements in intersecting set

Based on the similarity matrix, the disease sets were further classified into different clusters using Neighbor Joining (NJ) algorithm which is a bottom-up or agglomerative clustering method to identify similar group of elements in the data [5]. The Phylip tool [6] was used to implement the NJ method in our data.

### 2.3 Functional classification of data

The pathways information and transcription factor (TF) regulatory data were collected from the Molecular Signatures Database (MSigDB) [7]. Regulatory data is represented as a bipartite graph between a set of genes and transcription factors. Data for expression profiling of TFs in different brain tissues were obtained from GTEx portal web server [8].

### 2.4 Drug data analysis

A comprehensive search was made in DrugBank v4.0 (http://www.drugbank.ca/) database [9] to identify the known drugs related to our data set. Data was also obtained from a recent study by Rask-Andersen [10], to arrange the genes based on approved, investigational, not approved and withdrawn drug information.
