# Supplementary material for: Data on overlapping brain disorders and emerging drug targets in human Dopamine Receptors Interaction Network
Source: Data Brief. 2017 Apr 8;12:277–86. doi: 10.1016/j.dib.2017.04.001 (PMC5407500; doi:10.1016/j.dib.2017.04.001)
Supplement: Supplementary file 2 — Supplementary material [file mmc2.docx]

**TableS1: List of Significant Disease Terms (P < 0.05) Associated with DRIN**

| Sno. | **Disease** | **Disease Enrichment Score** | **Corrected**  **P-value** | **Rank** | **Percentage of Total DRIN** |
| --- | --- | --- | --- | --- | --- |
| 1 | Schizophrenia | 5.47049E-56 | 1.36734E-47 | 1 | 37.69063181 |
| 2 | Bipolar_Disorder | 9.61345E-54 | 2.40286E-45 | 2 | 25.708061 |
| 3 | Weight_Gain_Adverse_Event | 5.35861E-51 | 1.33937E-42 | 3 | 12.20043573 |
| 4 | Depressive_Disorder,_Major | 2.88805E-47 | 7.21861E-39 | 4 | 18.30065359 |
| 6 | depression | 4.47785E-47 | 1.11923E-38 | 5 | 21.1328976 |
| 5 | Mental_Disorders | 1.20342E-45 | 3.00793E-37 | 6 | 16.99346405 |
| 7 | Anxiety_Disorders | 1.46239E-44 | 3.65522E-36 | 7 | 15.46840959 |
| 8 | Unipolar_Depression | 1.92689E-43 | 4.81623E-35 | 8 | 15.90413943 |
| 9 | Weight_Gain | 4.51487E-43 | 1.12848E-34 | 9 | 13.07189542 |
| 10 | Depressive_Disorder | 2.14885E-42 | 5.371E-34 | 10 | 17.86492375 |
| 11 | Alcoholism | 4.56225E-40 | 1.14033E-31 | 11 | 21.1328976 |
| 12 | Mood_Disorders | 1.07834E-39 | 2.69529E-31 | 12 | 13.28976035 |
| 13 | Attention_Deficit_Disorder_with_Hyperactivity | 3.96962E-39 | 9.92197E-31 | 13 | 16.5577342 |
| 14 | Psychoses,_Substance-Induced | 2.89527E-37 | 7.23667E-29 | 14 | 7.625272331 |
| 15 | Substance_Withdrawal_Syndrome | 4.49001E-36 | 1.12227E-27 | 15 | 7.843137255 |
| 16 | Drug_abuse | 2.76949E-34 | 6.92229E-26 | 16 | 8.278867102 |
| 17 | Autistic_Disorder | 1.39983E-33 | 3.49884E-25 | 17 | 18.30065359 |
| 18 | substance_addiction | 1.53445E-33 | 3.83532E-25 | 18 | 7.189542484 |
| 19 | Obesity | 2.25002E-32 | 5.62388E-24 | 19 | 29.19389978 |
| 20 | Major_Affective_Disorder_2 | 6.19322E-32 | 1.54798E-23 | 20 | 7.407407407 |
| 21 | Alcohol_abuse | 1.01079E-31 | 2.52645E-23 | 21 | 7.843137255 |
| 22 | Cocaine-Related_Disorders | 2.95186E-31 | 7.3781E-23 | 22 | 8.278867102 |
| 23 | Nicotine_Dependence | 6.09449E-31 | 1.5233E-22 | 23 | 8.061002179 |
| 24 | Epilepsy | 1.52382E-30 | 3.80875E-22 | 24 | 15.90413943 |
| 25 | Alzheimer_Disease | 3.86104E-29 | 9.65057E-21 | 25 | 25.92592593 |
| 26 | Migraine_Disorders | 7.10364E-29 | 1.77554E-20 | 26 | 9.803921569 |
| 27 | as_if'_personality | 9.95448E-29 | 2.4881E-20 | 27 | 6.100217865 |
| 28 | Drug_Dependence | 9.95448E-29 | 2.4881E-20 | 28 | 6.100217865 |
| 29 | Seizures | 1.6688E-28 | 4.17112E-20 | 29 | 15.46840959 |
| 30 | Major_Affective_Disorder_4 | 6.23807E-28 | 1.55919E-19 | 30 | 6.535947712 |
| 31 | Major_Affective_Disorder_5 | 6.23807E-28 | 1.55919E-19 | 31 | 6.535947712 |
| 32 | Major_Affective_Disorder_6 | 6.23807E-28 | 1.55919E-19 | 32 | 6.535947712 |
| 33 | Major_Affective_Disorder_7 | 6.23807E-28 | 1.55919E-19 | 33 | 6.535947712 |
| 34 | Major_Affective_Disorder_8 | 6.23807E-28 | 1.55919E-19 | 34 | 6.535947712 |
| 35 | Major_Affective_Disorder_9 | 6.23807E-28 | 1.55919E-19 | 35 | 6.535947712 |
| 36 | Hypertension | 6.57371E-28 | 1.64308E-19 | 36 | 24.40087146 |
| 37 | Major_Affective_Disorder_1 | 1.60483E-27 | 4.01124E-19 | 37 | 6.535947712 |
| 38 | Psychotic_Disorders | 1.71915E-26 | 4.29697E-18 | 38 | 9.803921569 |
| 39 | Heroin_Dependence | 1.42422E-25 | 3.55981E-17 | 39 | 5.882352941 |
| 40 | Pain | 1.79652E-25 | 4.49037E-17 | 40 | 8.496732026 |
| 41 | Panic_Disorder | 4.40496E-25 | 1.10101E-16 | 41 | 6.97167756 |
| 42 | Marijuana_Abuse | 7.71692E-25 | 1.92883E-16 | 42 | 6.535947712 |
| 43 | Pituitary_Diseases | 1.79694E-23 | 4.49142E-15 | 43 | 13.07189542 |
| 44 | opiate_addiction | 5.05142E-23 | 1.26259E-14 | 44 | 4.357298475 |
| 45 | Hypotension | 9.02201E-23 | 2.25503E-14 | 45 | 5.446623094 |
| 46 | psychosis | 4.21199E-22 | 1.05278E-13 | 46 | 8.061002179 |
| 47 | Amphetamine-Related_Disorders | 7.8987E-22 | 1.97426E-13 | 47 | 5.882352941 |
| 48 | Stress_(Psychology) | 8.14005E-22 | 2.03459E-13 | 48 | 33.11546841 |
| 49 | Hyperalgesia | 2.71829E-21 | 6.7943E-13 | 49 | 7.407407407 |
| 50 | Heroin_abuse | 3.33807E-21 | 8.34344E-13 | 50 | 3.48583878 |
| 51 | central_neuroblastoma | 3.49593E-21 | 8.73799E-13 | 51 | 22.0043573 |
| 52 | Neuroblastoma | 4.20165E-21 | 1.05019E-12 | 52 | 23.74727669 |
| 53 | Asthma | 7.86374E-21 | 1.96552E-12 | 53 | 21.56862745 |
| 54 | Borderline_Personality_Disorder | 9.46116E-21 | 2.3648E-12 | 54 | 7.407407407 |
| 55 | Sleep_Disorders | 9.67451E-21 | 2.41812E-12 | 55 | 5.664488017 |
| 56 | Inflammation | 1.59408E-20 | 3.98436E-12 | 56 | 14.37908497 |
| 57 | Personality_Disorders | 2.17753E-20 | 5.44268E-12 | 57 | 3.703703704 |
| 58 | Anorexia_Nervosa | 2.37499E-20 | 5.93623E-12 | 58 | 6.100217865 |
| 59 | Schizoaffective_Disorder | 2.93652E-19 | 7.33976E-11 | 59 | 5.010893246 |
| 60 | Antisocial_behavior | 6.94168E-19 | 1.73506E-10 | 60 | 3.921568627 |
| 61 | Catalepsy | 8.04192E-19 | 2.01006E-10 | 61 | 3.267973856 |
| 62 | Dyskinesias | 1.13936E-18 | 2.8478E-10 | 62 | 4.575163399 |
| 63 | Cocaine_Dependence | 1.26039E-18 | 3.15032E-10 | 63 | 4.357298475 |
| 64 | Parkinson_Disease | 3.01706E-18 | 7.54107E-10 | 64 | 13.7254902 |
| 65 | Epilepsy,_Temporal_Lobe | 3.24554E-18 | 8.11215E-10 | 65 | 6.97167756 |
| 66 | Substance_Abuse | 3.86397E-18 | 9.65792E-10 | 66 | 3.921568627 |
| 67 | Orofacial_dyskinesia | 1.14111E-17 | 2.85219E-09 | 67 | 4.575163399 |
| 68 | Stress_Disorders,_Post-Traumatic | 1.42688E-17 | 3.56645E-09 | 68 | 4.793028322 |
| 69 | Pheochromocytoma | 1.44697E-17 | 3.61667E-09 | 69 | 7.843137255 |
| 70 | personality_traits | 2.02164E-17 | 5.05304E-09 | 70 | 3.050108932 |
| 71 | Behavior,_Addictive | 2.51619E-17 | 6.28916E-09 | 71 | 6.535947712 |
| 72 | Hypertension,_Essential | 4.78017E-17 | 1.19479E-08 | 72 | 8.496732026 |
| 73 | Withdrawal_sign_or_symptom | 5.03237E-17 | 1.25783E-08 | 73 | 3.050108932 |
| 74 | Bipolar_I_disorder | 1.01392E-16 | 2.53428E-08 | 74 | 4.357298475 |
| 75 | PANIC_DISORDER_1 | 1.65635E-16 | 4.14E-08 | 75 | 4.575163399 |
| 76 | Tourette_Syndrome | 2.34453E-16 | 5.86009E-08 | 76 | 5.010893246 |
| 77 | Headache | 2.72935E-16 | 6.82195E-08 | 77 | 3.921568627 |
| 78 | Neuroticism | 3.0736E-16 | 7.6824E-08 | 78 | 4.139433551 |
| 79 | Heart_Septal_Defects,_Atrial | 3.54259E-16 | 8.85462E-08 | 79 | 7.407407407 |
| 80 | Hyperkinesis | 3.99619E-16 | 9.9884E-08 | 80 | 3.267973856 |
| 81 | Schizophrenics | 4.10601E-16 | 1.02629E-07 | 81 | 4.575163399 |
| 82 | Tardive_Dyskinesia | 4.41703E-16 | 1.10403E-07 | 82 | 4.139433551 |
| 83 | Atherosclerosis | 5.14482E-16 | 1.28594E-07 | 83 | 17.4291939 |
| 84 | Alcohol_withdrawal_syndrome | 7.32744E-16 | 1.83148E-07 | 84 | 3.267973856 |
| 85 | Memory_Disorders | 9.03347E-16 | 2.2579E-07 | 85 | 3.921568627 |
| 86 | Diabetes_Mellitus,_Type_2 | 1.01219E-15 | 2.52995E-07 | 86 | 22.44008715 |
| 87 | Bradycardia | 1.11192E-15 | 2.77923E-07 | 87 | 3.050108932 |
| 88 | Pulmonary_Disease,_Chronic_Obstructive | 1.28595E-15 | 3.21421E-07 | 88 | 13.7254902 |
| 89 | Substance-Related_Disorders | 5.82309E-15 | 1.45547E-06 | 89 | 6.100217865 |
| 90 | Dyskinesia,_Drug-Induced | 1.04801E-14 | 2.61948E-06 | 90 | 3.48583878 |
| 91 | Fatigue_Syndrome,_Chronic | 2.36624E-14 | 5.91436E-06 | 91 | 3.48583878 |
| 92 | Neuroendocrine_Tumors | 2.8166E-14 | 7.04002E-06 | 92 | 6.97167756 |
| 93 | Nervous_System_Diseases | 3.86331E-14 | 9.65626E-06 | 93 | 9.368191721 |
| 94 | Diabetes_Mellitus,_Experimental | 5.12553E-14 | 1.28111E-05 | 94 | 9.586056645 |
| 95 | Bronchopulmonary_Dysplasia | 5.42643E-14 | 1.35632E-05 | 95 | 6.100217865 |
| 96 | Heart_Failure | 7.11652E-14 | 1.77876E-05 | 96 | 15.03267974 |
| 97 | Neoplasms | 1.32278E-13 | 3.30625E-05 | 97 | 23.52941176 |
| 98 | SUBSTANCE_USE_DISORDER | 1.33656E-13 | 3.3407E-05 | 98 | 2.832244009 |
| 99 | Prenatal_Exposure_Delayed_Effects | 1.50195E-13 | 3.75409E-05 | 99 | 3.48583878 |
| 100 | Mixed_anxiety_and_depressive_disorder | 2.29728E-13 | 5.74201E-05 | 100 | 2.832244009 |
| 101 | Impulse_Control_Disorders | 3.35867E-13 | 8.39493E-05 | 101 | 1.960784314 |
| 102 | Anorexia | 4.56064E-13 | 0.000113992 | 102 | 3.921568627 |
| 103 | Autism_spectrum_disorders | 5.89343E-13 | 0.000147305 | 103 | 6.100217865 |
| 104 | Amnesia | 7.64795E-13 | 0.000191159 | 104 | 3.48583878 |
| 105 | Obsessive-Compulsive_Disorder | 8.5406E-13 | 0.00021347 | 105 | 4.139433551 |
| 106 | Chronic_Pain | 9.139E-13 | 0.000228427 | 106 | 3.050108932 |
| 107 | Status_Epilepticus | 9.336E-13 | 0.000233351 | 107 | 5.22875817 |
| 108 | Metabolic_Syndrome_X | 1.05962E-12 | 0.000264849 | 108 | 10.67538126 |
| 109 | Antisocial_Personality_Disorder | 1.60196E-12 | 0.000400406 | 109 | 2.832244009 |
| 110 | Malignant_neoplasm_of_prostate | 1.67392E-12 | 0.000418392 | 110 | 28.32244009 |
| 111 | Eating_Disorders | 2.07051E-12 | 0.00051752 | 111 | 3.703703704 |
| 112 | Stress,_Psychological | 2.4737E-12 | 0.000618295 | 112 | 3.48583878 |
| 113 | prostate_carcinoma | 2.74327E-12 | 0.000685675 | 113 | 27.01525054 |
| 114 | Prolactinoma | 3.07311E-12 | 0.000768116 | 114 | 3.921568627 |
| 115 | alcohol_use_disorder | 3.78418E-12 | 0.000945846 | 115 | 2.832244009 |
| 116 | Nausea | 3.78418E-12 | 0.000945846 | 116 | 2.832244009 |
| 117 | Endometriosis | 5.06566E-12 | 0.001266151 | 117 | 11.11111111 |
| 118 | Parkinsonian_Disorders | 6.72718E-12 | 0.001681443 | 118 | 5.664488017 |
| 119 | Insulinoma | 6.87684E-12 | 0.001718851 | 119 | 5.010893246 |
| 120 | Ischemia | 8.67207E-12 | 0.002167565 | 120 | 10.45751634 |
| 121 | Alcohol-Related_Disorders | 1.12889E-11 | 0.002821644 | 121 | 1.960784314 |
| 122 | Delusions | 1.22847E-11 | 0.003070529 | 122 | 2.178649237 |
| 123 | Brain_Injuries | 1.79383E-11 | 0.004483629 | 123 | 5.882352941 |
| 124 | Prostatic_Neoplasms | 1.8876E-11 | 0.004718015 | 124 | 18.73638344 |
| 125 | Cocaine_Abuse | 2.74823E-11 | 0.006869135 | 125 | 1.960784314 |
| 126 | Substance_use | 2.74823E-11 | 0.006869135 | 126 | 1.960784314 |
| 127 | Hyperphagia | 2.8386E-11 | 0.007095018 | 127 | 3.050108932 |
| 128 | Affective_Disorders,_Psychotic | 3.73416E-11 | 0.009333452 | 128 | 1.74291939 |
| 129 | Sexual_abuse | 3.73416E-11 | 0.009333452 | 129 | 1.74291939 |
| 130 | Weight_Loss | 3.81099E-11 | 0.009525492 | 130 | 3.050108932 |
| 131 | body_mass | 4.31453E-11 | 0.010784084 | 131 | 3.921568627 |
| 132 | Diabetes_Mellitus | 4.3341E-11 | 0.010832993 | 132 | 17.64705882 |
| 133 | Bulimia | 6.04763E-11 | 0.015115916 | 133 | 2.396514161 |
| 134 | Methamphetamine_abuse | 6.11694E-11 | 0.015289144 | 134 | 1.960784314 |
| 135 | Pneumonia | 6.58198E-11 | 0.016451515 | 135 | 7.625272331 |
| 136 | Bronchiolitis,_Viral | 7.19147E-11 | 0.017974917 | 136 | 5.664488017 |
| 137 | Dermatitis,_Atopic | 7.30958E-11 | 0.01827013 | 137 | 8.496732026 |
| 138 | Hypertension,_Pulmonary | 8.41105E-11 | 0.021023228 | 138 | 6.753812636 |
| 139 | Adenoma | 8.57728E-11 | 0.021438725 | 139 | 13.28976035 |
| 140 | Hyperglycemia | 1.06493E-10 | 0.026617616 | 140 | 7.843137255 |
| 141 | Osteochondritis_Dissecans | 1.12893E-10 | 0.028217429 | 141 | 3.267973856 |
| 142 | Primary_malignant_neoplasm | 1.74411E-10 | 0.04359354 | 142 | 43.1372549 |
| 143 | Fatigue | 1.9088E-10 | 0.047709911 | 143 | 4.357298475 |

**Disease:** represented disease terms identified from DisGeNet database

**Disease Enrichment Score:** calculated by two-sided hypergeometric test

**Corrected P-value:** bonferroni correction (p ≤ 0.05)

**Rank:** list of significant disease association in our data in descending order

**Percentage of Total DRIN:** Number of genes associated with particular disease term in DRIN
